# Supplementary material for: Metabolic Profiling of the Oil of Sesame of the Egyptian Cultivar ‘Giza 32’ Employing LC-MS and Tandem MS-Based Untargeted Method
Source: Foods. 2021 Feb 2;10(2):298. doi: 10.3390/foods10020298 (PMC7913063; doi:10.3390/foods10020298)
Supplement: Supplementary file 1 [file foods-10-00298-s001.pdf]

foods

Gradient Elution and operating conditions by RP-HPLC-DAD-ESI-QTOF-MS/MS

The gradient elution was conducted according [10,22] with two mobile phases, 0.5% acetic acid, *v*/*v* (phase A) and acetonitrile (phase B), with a constant flow rate of 0.5 mL min^{-1}. The gradient program was as follows: 0 min 99% A and 1% B, 5.50 min 93% A and 7% B, 11 min 86% A and 14% B, 17.5 min 76% A and 24% B, 22.50 min 60% A and 40% B, 27.50 min 0% A and 100% B, 28.5 min 0% A and 100% B, 29.5 min. For column equilibration, initial conditions were finally maintained for 5.50 min. (total run 35 min). The injection volume was 15 µl. Briefly, the operating conditions were: drying nitrogen gas temperature 325 °C with a flow of 10 L min^{-1}; nebulizer pressure 20 psig; sheath gas temperature 400 °C with a flow 12 L min^{-1}; capillary voltage 4000 V; nozzle voltage 500 V; fragmentor voltage 130 V; skimmer voltage 45 V; octapole radiofrequency voltage 750 V.

Data acquisition (2.5 Hz) in profile mode was governed via MassHunter Workstation software (Agilent technologies). The spectra were acquired in the negative ionization mode, over a mass-to-charge (m/z) range from 70 to 1100. The detection window was set to 100 ppm. Reference mass correction on each sample was performed with a continuous infusion of Agilent TOF biopolymer analysis mixture containing trifluoroacetic acid ammonium salt (m/z 112.9856) and hexakis (1H, 1H, 3H-tetrafluoropropoxy) phosphazine (m/z 980.0164 corresponding to the acetic adduct).

Table S1. Phenolic compounds characterized in SG32 oil.

Number	RT (min)	Experimental *m/z*[a] [M-H]⁻	Theoretical mass (M)	Molecular formula	Error (ppm)	Error (mDa)	Score	Main fragments	DBE	UV (nm)	Proposed compound	Subclass	Species	Family	Reference	Oil	Cake
9	4.70	151.0402	152.0473	$C_8H_8O_3$	−0.26	0.0	99	123.0450, 122.0372	5	267	Vanillin	Phenolic aldehyde	*Olea europaea*	Oleaceae	[47]	+	-
16	7.31	169.0140	170.0215	$C_7H_6O_5$	1.25	0.20	87	125.0247	5	N.D.	Gallic acid*	Hydroxybenzoic acid	*Sesamum indicum*	Pedaliaceae	[10]	+	+
20	8.74	137.0243	138.0316	$C_7H_6O_3$	0.34	0.05	94	N.D.	5	N.D.	Sesamol	Phenol derivative	*Sesamum indicum*	Pedaliaceae	[10]	+	+
21	9.19	147.0447	148.0524	$C_9H_8O_2$	3.0	0.5	96	103.0556	6	N.D.	Cinnamic acid	Hydroxycinnamic acid	*Sesamum indicum*	Pedaliaceae	[10]	+	+
24	10.64	329.0879	330.0951	$C_{14}H_{18}O_9$	−0.24	−0.08	99	167.0347, 152.0114, 123.0450. 108.0218	6	255, 286	Vanillic acid hexoside	Hydroxybenzoic acid	*Sesamum indicum*	Pedaliaceae	[10]	+	+
25	11.33	153.0192	154.0266	$C_7H_6O_4$	1.02	0.16	99	137.0244, 109.0293, 108.0213	5	258, 288 sh	3,4-Dihydroxybenzoic acid* (protocatechuic acid)	Hydroxybenzoic acid	*Sesamum indicum*	Pedaliaceae	[10]	+	+
26	11.83	359.0978	360.1057	$C_{15}H_{20}O_{10}$	1.2	0.4	97	197.0474, 182.0213, 153.0544, 138.0318	6	N.D.	Syringic acid hexoside	Hydroxybenzoic acid	*Sesamum indicum*	Pedaliaceae	[10]	+	+
27	12.47	325.0932	326.1002	$C_{15}H_{18}O_8$	0.4	0.1	94	163.0398, 119.0502	7	287	*p*-Coumaric acid hexoside I	Hydroxycinnamic acid	*Cicer arietinum/ Sesamum indicum*	Fabaceae /Pedaliaceae	[10,21]	+	+
28	12.72	325.0931	326.1002	$C_{15}H_{18}O_8$	−0.5	−0.16	99	163.0400, 119.0502	7	288	*p*-Coumaric acid hexoside II	Hydroxycinnamic acid	*Cicer arietinum/ Sesamum indicum*	Fabaceae /Pedaliaceae	[10,21]	+	+
30	13.95	355.1035	356.1107	$C_{16}H_{20}O_9$	−0.1	−0.03	99	193.0505, 178.0271, 149.0607	7	279	Ferulic acid hexoside I	Hydroxycinnamic acid	*Cicer arietinum/ Sesamum indicum*	Fabaceae /Pedaliaceae	[10,21]	+	+
31	14.01	355.1037	356.1107	$C_{16}H_{20}O_9$	−0.69	−0.25	100	N.D.	7	282	Ferulic acid hexoside II	Hydroxycinnamic acid	*Cicer arietinum/ Sesamum indicum*	Fabaceae /Pedaliaceae	[10,21]	+	+

32	14.36	137.0240	138.0317	$C_7H_6O_3$	−1.1	−0.15	99	119.0142, 109.0293, 108.0218, 93.0344, 92.0269	5	273	Hydroxybenzoic acid	Hydroxybenzoic acid	*Sesamum indicum*	Pedaliaceae	[10]	+	+
33	14.49	151.0401	152.0473	$C_8H_8O_3$	−0.88	−0.13	97	137.0245, 107.0506	5	237, 274	Hydroxybenzoic acid methyl ester	Hydroxybenzoic acid			[25]	+	+
35	14.99	353.0879	354.0951	$C_{16}H_{18}O_9$	−0.45	−0.16	99	191.0559,179.0344, 173.0457, 161.0246, 135.0452	8	238, 279	Caffeoylquinic acid I* (Chlorogenic acid)	Hydroxycinnamic acid	*Cicer arietinum*/ *Sesamum indicum*	Fabaceae /Pedaliaceae	[10,21]	+	+
36	15.10	385.1137	386.1213	$C_{17}H_{22}O_{10}$	−0.6	−0.23	88	223.0611, 208.0373, 193.0141, 179.0703, 164.0472, 149.0242	7	290	Sinapic acid hexoside	Hydroxycinnamic acid	*Cicer arietinum*/ *Sesamum indicum*	Fabaceae /Pedaliaceae	[10,21]	+	+
37	15.53	325.0928	326.1002	$C_{15}H_{18}O_8$	−0.15	−0.05	98	163.0397, 119.0503	7	277	*p*-Coumaric acid hexoside III	Hydroxycinnamic acid	*Cicer arietinum*/ *Sesamum indicum*	Fabaceae /Pedaliaceae	[10,21]	+	+
38	16.61	325.0929	326.1002	$C_{15}H_{18}O_8$	−1.1	−0.36	89	N.D.	7	N.D.	*p*-Coumaric acid hexoside IV	Hydroxycinnamic acid	*Cicer arietinum*/ *Sesamum indicum*	Fabaceae /Pedaliaceae	[10,21]	+	+
39	16.68	355.1033	356.1107	$C_{16}H_{20}O_9$	0.5	0.18	100	193.0500, 178.0272, 149.0609	7	282	Ferulic acid hexoside III	Hydroxycinnamic acid	*Cicer arietinum*/ *Sesamum indicum*	Fabaceae /Pedaliaceae	[10,21]	+	+
40	16.73	167.0346	168.0426	$C_8H_8O_4$	1.29	0.22	98	152.0144, 123.0449, 108.0251	5	258	Vanillic acid*	Hydroxybenzoic acid	*Sesamum indicum*	Pedaliaceae	[10]	+	+
41	17.11	593.1510	594.1585	$C_{27}H_{30}O_{15}$	0.3	0.15	99	503.1188, 473.1085, 443.0981, 413.0872, 383.0767, 353.0667, 135.0457, 119.0357, 117.0367	13	267, 320	Luteolin *C*-deoxyhexoside *C*-hexoside I	Flavone	*Sesamum indicum*	Pedaliaceae	[10]	+	+
42	17.23	353.0876	354.0951	$C_{16}H_{18}O_9$	0.3	0.1	96	191.0556, 179.0327, 173.0450, 161.0233, 135.0445	8	274	Caffeoylquinic acid II	Hydroxycinnamic acid	*Cicer arietinum*/ *Sesamum indicum*	Fabaceae /Pedaliaceae	[10,21]	+	+

43	17.36	593.1590	594.1585	$C_{27}H_{30}O_{15}$	0.3	0.15	99	533.1285, 503.1193, 473.1085, 443.0989, 413.0873, 383.0771, 353.0665	13	270, 325	Luteolin *C*-deoxyhexoside *C*-hexoside II	Flavone	*Sesamum indicum*	Pedaliaceae	[10]	+	+
44	17.34	197.0453	198.05282	$C_9H_{10}O_5$	0.6	0.1	97	N.D.	5		Syringic acid*	Hydroxybenzoic acid	*Cicer arietinum/ Sesamum indicum*	Fabaceae /Pedaliaceae	[10,21]	+	−
45	17.47	355.1034	356.1107	$C_{16}H_{20}O_9$	0.4	0.16	96	N.D.	7	287	Ferulic acid hexoside IV	Hydroxycinnamic acid	*Cicer arietinum/ Sesamum indicum*	Fabaceae /Pedaliaceae	[10,21]	+	−
46	17.78	289.0718	290.0790	$C_{15}H_{14}O_6$	0.79	0.02	93	253.0334, 245.1390, 217.0027, 131.0712, 123.0450	9	N.D.	(-)-Epicatechin*	Flavan-3-ol	*Sesamum indicum*	Pedaliaceae	[10]	+	+
47	17.78	531.1719	532.1792	$C_{23}H_{32}O_{14}$	−0.53	−0.28	89	179.0140, 165.0554, 150.0317	8	283	Sinapic acid deoxyhexoside hexoside	Hydroxycinnamic acid	*Sesamum indicum*	Pedaliaceae	[10]	+	+
48	17.96	681.2400	682.2473	$C_{32}H_{42}O_{16}$	0.54	0.37	100	357.1302, 151.0384	12	242, 275	Pinoresinol dihexoside I	Lignan	*Sesamum indicum*	Pedaliaceae	[10]	+	+
49	18.21	563.1411	564.1479	$C_{26}H_{28}O_{14}$	−0.57	−0.32	99	545.1297, 503.1189, 473.1087, 443.0979, 413.0872, 383.0771, 353.0664, 117.0343	13	274, 330	Apigenin *C*-pentoside *C*-hexoside I	Flavone	*Sesamum indicum*	Pedaliaceae	[10]	+	+
50	18.27	121.0293	122.0368	$C_7H_6O_2$	1.20	0.15	100	92.0269	5	278	Benzoic acid	Hydroxybenzoic acid	*Sesamum indicum*	Pedaliaceae	[10]	+	+
51	18.63	563.1405	564.1479	$C_{26}H_{28}O_{14}$	0.08	0.05	99	545.1323, 503.1179, 473.1083, 443.0976, 413.0871, 383.0767, 353.0666, 117.0335	13	272, 327	Apigenin *C*-pentoside *C*-hexoside II	Flavone	*Sesamum indicum*	Pedaliaceae	[10]	+	+
52	18.89	563.1408	564.1479	$C_{26}H_{28}O_{14}$	0.08	0.05	99	545.1298, 503.1191, 473.1087, 443.0976, 413.0878, 383.0769, 353.0666, 117.0336	13	269, 331	Apigenin *C*-pentoside *C*-hexoside III	Flavone	*Sesamum indicum*	Pedaliaceae	[10]	+	+
53	18.87	447.0935	448.1006	$C_{21}H_{20}O_{11}$	−0.46	−0.21	99	327.0515, 179.0141, 135.0447	12	268, 325	Luteolin *C*-hexoside I	Flavone	*Sesamum indicum*	Pedaliaceae	[10]	+	+

54	19.50	447.0930	448.1006	$C_{21}H_{20}O_{11}$	0.28	0.21	92	327.0539, 179.0138, 135.0450	12	N.D.	Luteolin *C*-hexoside II	Flavone	*Sesamum indicum*	Pedaliaceae	[10]	+	+
55	20.34	151.0400	152.0473	$C_8H_8O_3$	−0.28	0.0	98	136.0167, 92.0269	5	256	Methoxybenzoic acid I	Hydroxybenzoic acid			[25]	+	−
56	20.40	163.0399	164.0473	$C_9H_8O_3$	0.47	0.08	97	N.D.	6	N.D.	*p*-Coumaric acid*	Hydroxycinnamic acid	*C. arietinum*	Fabaceae	[21]	+	+
57	20.46	609.1472	610.1534	$C_{27}H_{30}O_{16}$	−1.41	−0.86	96	300.0267, 151.0033	13	255, 355	Quercetin 3-*O*-rutinoside (rutin)*	Flavonol	*Cicer arietinum*/ *Sesamum indicum*	Fabaceae /Pedaliaceae	[10,21]	+	+
58	20.59	593.1510	594.1585	$C_{27}H_{30}O_{15}$	0.3	0.16	99	447.0897, 285.0396, 133.0281	13	267, 320	Luteolin deoxyhexoside hexoside	Flavone	*Sesamum indicum*	Pedaliaceae	[10]	+	−
59	20.93	300.9988	302.00627	$C_{14}H_6O_8$	0.82	0.25	95	N.D.	12	N.D.	Ellagic acid*	Hexahydroxydiphenic acid dilactone	*Sesamum indicum*	Pedaliaceae	[6]	+	−
60	21.06	463.0874	464.09548	$C_{21}H_{20}O_{12}$	0.25	0.12	85	300.9980, 151.0030	12	N.D.	Quercetin 3-*O*-β-D-galactopyranoside*	Flavonol	*Cicer arietinum*/ *Sesamum indicum*	Fabaceae /Pedaliaceae	[10,21]	+	+
61	21.18	623.1972	624.2054	$C_{29}H_{36}O_{15}$	1.52	0.95	94	461.1649, 387.1502, 315.1074, 297.0957, 179.0347, 161.0239, 153.0543, 135.0447, 113.0233	12	N.D.	Verbascoside	Hydroxycinnamic acid	*Sesamum indicum*	Pedaliaceae	[10]	+	+
62	21.18	681.2402	682.2473	$C_{32}H_{42}O_{16}$	−0.12	−0.08	99	519.1525, 357.1333, 179.0529,151.0382, 149.0467	12	N.D.	Pinoresinol dihexoside II	Lignan	*Sesamum indicum*	Pedaliaceae	[10]	+	+
63	21.30	463.0883	464.09548	$C_{21}H_{20}O_{12}$	−0.02	−0.01	99	301.0324, 300.0248 271.02228, 255.0276, 178.9974, 151.0027, 136.0172, 135.0447	12	250, 352	Quercetin 3-*O*-β-D-glucopyranoside*	Flavonol	*Cicer arietinum*/ *Sesamum indicum*	Fabaceae /Pedaliaceae	[10,21]	+	+
64	21.36	447.0936	448.1006	$C_{21}H_{20}O_{11}$	−0.84	−0.38	97	285.0402,151.0026, 133.0284	12	N.D.	Luteolin 7-*O*-β-D-glucopyranoside*	Flavone	*Sesamum indicum*	Pedaliaceae	[10]	+	+
65	21.87	575.1189	576.1268	$C_{30}H_{24}O_{12}$	0.51	0.30	93	N.D.	19	N.D.	Procyanidin A2*	Proanthocyanidin	*Vaccinium macrocarpon*	Ericaceae	[37]	+	+

66	21.79	1017.3111	1018.3165	$C_{44}H_{58}O_{27}$	−1.71	−1.74	97	855.2573, 693.2019, 369.0973, 323.0977, 221.0642, 219.0663, 179.0559, 161.0452, 149.0451, 143.0349	16	280	Sesaminol tetrahexoside I	Lignan	*Sesamum indicum*	Pedaliaceae	[10]	+	+
67	22.27	1017.3095	1018.3165	$C_{44}H_{58}O_{27}$	−0.15	−0.15	98	855.2556, 693.2026, 369.0971, 323.0973, 221.0682, 179.0555, 161.0459, 149.0443, 143.0342	16	280	Sesaminol tetrahexoside II	Lignan	*Sesamum indicum*	Pedaliaceae	[10]	+	+
68	22.39	593.1504	594.1585	$C_{27}H_{30}O_{15}$	0.84	0.50	96	N.D.	13	N.D.	Kaempferol 3-*O*-rutinoside* [Kaempferol 3-*O*-(α-L-rhamnopyranosyl-(1→6)-β-D-glucopyranoside)]	Flavonol	*Cicer arietinum*	Fabaceae	[21]	+	−
69	22.52	841.2775	842.2857	$C_{38}H_{50}O_{21}$	−0.04	−0.04	99	679.2225, 485.1504, 355.1176, 323.0978, 221.0665, 179.0548, 161.0454, 149.0450, 143.0352, 121.0288, 89.0245	14	286	Xanthoxylol trihexoside	Lignan	*Sesamum indicum*	Pedaliaceae	[10]	+	+
70	22.54	163.0404	164.0473	$C_9H_8O_3$	−1.97	−0.32	84	N.D.	6	284	*m*-Coumaric acid*	Hydroxycinnamic acid	*Sesamum indicum*	Pedaliaceae	[10]	+	+
71	22.60	193.0505	194.0579	$C_{10}H_{10}O_4$	0.5	0.1	100	178.0270, 134.0371, 119.0503	6	230, 282, 310	Ferulic acid*	Hydroxycinnamic acid	*Sesamum indicum*	Pedaliaceae	[10]	+	+
72	22.73	855.2582	856.2637	$C_{38}H_{48}O_{22}$	−1.18	−1.55	97	693.2036, 485.1494, 369.0963, 323.0999, 221.0663, 179.0556, 161.0456, 149.0446, 143.0346, 119.0348	15	278	Sesaminol trihexoside I	Lignan	*Sesamum indicum*	Pedaliaceae	[10]	+	+
73	23.18	609.1822	610.1898	$C_{28}H_{34}O_{15}$	0.49	0.3	98	447.1293, 301.0713, 259.0811, 175.0023, 151.0031	12	281	Hesperetin hexoside deoxyhexoside	Flavanone		Several families	[26]	+	−
74	23.20	447.0943	448.1006	$C_{21}H_{20}O_{11}$	−2.07	−0.93	97	285.0404, 135.0452, 127.0764	12	N.D.	Kaempferol 3-*O*-β-D-glucopyranoside*	Flavonol	*Cicer arietinum*	Fabaceae	[21]	+	−

75	23.30	855.2572	856.2637	$C_{38}H_{48}O_{22}$	−0.62	−0.53	98	693.2060, 485.1514, 369.0970, 323.0983, 221.0667, 179.0560, 161.0453, 149.0452, 143.0352, 119.0351	15	277	Sesaminol trihexoside II	Lignan	*Sesamum indicum*	Pedaliaceae	[10]	+	+
76	23.49	855.2568	856.2637	$C_{38}H_{48}O_{22}$	−0.28	−0.24	100	693.2029, 485.1508, 369.0981, 323.0980, 221.0660, 179.0563, 161.0457, 149.0451, 143.0351, 119.0349	15	287	Sesaminol trihexoside III	Lignan	*Sesamum indicum*	Pedaliaceae	[10]	+	+
78	24.21	871.2521	872.2586	$C_{38}H_{48}O_{23}$	−0.7	−0.61	95	709.1983, 691.1938, 485.1527, 385.0925, 323.0985, 221.0664, 179.0556, 161.0447, 143.0354, 137.0245, 119.0354, 89.0245	15	277	Hydroxysesamolin tri-hexoside	Lignan	*Sesamum indicum*	Pedaliaceae	[10]	+	+
79	24.27	539.1775	540.1843	$C_{25}H_{32}O_{13}$	−0.8	−0.43	99	377.1240, 359.1192, 333.0842, 327.0880, 275.0918, 223.0641, 209.0455, 191.0349, 179.0697, 171.0293, 161.0473, 153.0559, 127.0400	10	235, 273	**Sinapoyl-3-dehydro-shikimic acid hexoside I**	Hydroxycinnamic acid				+	−
80	24.24	135.0451	136.0525	$C_8H_8O_2$	−0.17	−0.02	85	77.0400	5	273	Methyl benzoic acid	Hydroxybenzoic acid	*Sesamum indicum*	Pedaliaceae	[10]	+	−
81	24.43	679.2248	680.2319	$C_{32}H_{40}O_{16}$	−0.35	−0.24	99	517.2748, 485.1429, 355.1176, 323.0964, 221.0661, 179.0566, 161.0453, 149.0449, 143.0342, 121.0288, 89.0244	13	282	**Xanthoxylol dihexo-side**	Lignan				+	−
82	24.76	539.1775	540.1843	$C_{25}H_{32}O_{13}$	0.52	0.28	99	333.0854, 327.0889, 223.0609 209.0446, 191.0340, 171.0299, 161.0482, 153.0537, 127.0399	10	230, 277	**Sinapoyl-3-dehydro-shikimic acid hexoside II**	Hydroxycinnamic acid				+	−

83	24.82	161.0249	162.0322	$C_9H_6O_3$	−3.38	−0.19	76	N.D.	7	N.D.	7-Hydroxycoumarin (umbelliferone)*	Coumarin	*Sesamum indicum*	Pedaliaceae	[10]	+	+
84	24.97	151.0400	152.0473	$C_8H_8O_3$	−0.13	0.0	100	136.0167, 92.0270	5	N.D.	Methoxybenzoic acid II	Hydroxybenzoic acid			[25]	+	−
85	26.45	285.0408	286.0477	$C_{15}H_{10}O_6$	−1.18	−0.34	99	227.1288, 135.0450	11	287, 325	Luteolin*	Flavone	*Sesamum indicum*	Pedaliaceae	[10]	+	+
86	28.32	271.0614	272.0685	$C_{15}H_{12}O_5$	−0.73	−0.2	99	N.D.	10	N.D.	Naringenin*	Flavanone	*Cicer arietinum/ Sesamum indicum*	Fabaceae /Pedaliaceae	[10,21]	+	+

[a] Detected ions were $[M-H]^-$.* Identification confirmed by comparison with standards; N.D., below 5 mAU or masked by compound with higher signal. Isomers are denoted with letter codes I, II, etc. Compounds in bold letter indicate new proposed structures.

Table 2. Non-phenolic compounds characterized in Sg32 oil.

Number	RT (min)	Experimental *m*/*z*[a] [M-H]-	Theoretical mass (M)	Molecular formula	Error (ppm)	Error (mDa)	Score	Main fragments	DBE	UV (nm)	Proposed compound	Subclass	Species	Family	Reference	Oil	Cake
1	2.65	195.0506	196.0583	$C_6H_{12}O_7$	2.33	0.046	98	165.0398	1	N.D.	Gluconic/ galactonic acid	Organic acid	*Cicer arietinum*/ *Sesamum indicum*	Fabaceae /Pedaliaceae	[21,10]	+	+
2	3.09	191.0201	192.027	$C_6H_8O_7$	−1.72	−0.33	99	173.0094, 111.0089	3	N.D.	Citric acid I	Organic acid	*Cicer arietinum*/ *Sesamum indicum*	Fabaceae /Pedaliaceae	[21,10]	+	+
3	3.15	133.0142	134.0215	$C_4H_6O_5$	0.29	0.04	99	115.0039	2	N.D.	Malic acid I	Organic acid	*Cicer arietinum*/ *Sesamum indicum*	Fabaceae /Pedaliaceae	[21,10]	+	+
4	3.40	133.0141	134.0215	$C_4H_6O_5$	1.37	0.18	99	115.004	2	N.D.	Malic acid II	Organic acid	*Cicer arietinum*/ *Sesamum indicum*	Fabaceae /Pedaliaceae	[21,10]	+	+
5	4.02	191.0203	192.027	$C_6H_8O_7$	−2.91	−0.56	98	173.0096, 111.0092	3	N.D.	Citric acid II	Organic acid	*Cicer arietinum*/ *Sesamum indicum*	Fabaceae /Pedaliaceae	[21,10]	+	+
6	4.08	128.0358	129.0426	$C_5H_7NO_3$	−2.98	−0.38	96	111.0092	3	N.D.	Pyroglutamic acid I	Amino acid		Several families	[26]	+	+
7	4.27	191.0191	192.027	$C_6H_8O_7$	3.25	0.62	97	173.0081, 111.0088	3	N.D.	Citric acid III	Organic acid	*Cicer arietinum*/ *Sesamum indicum*	Fabaceae /Pedaliaceae	[21,10]	+	+
8	4.58	128.0353	129.0426	$C_5H_7NO_3$	−0.83	−0.11	98	111.0092	3	N.D.	Pyroglutamic acid II	Amino acid		Several families	[26]	+	+
10	5.01	130.0869	131.0949	$C_6H_{13}NO_2$	3.76	0.49	96	112.9856	1	N.D.	Leucine/Isoleucine I	Amino acid	*Cicer arietinum*/ *Sesamum indicum*	Fabaceae /Pedaliaceae	[21,10]	+	+
11	5.45	117.0193	118.0266	$C_4H_6O_4$	0	0	98	73.0298	2	N.D.	Succinic acid	Organic acid	*C. arietinum*	Fabaceae	[21]	+	+
12	5.51	130.087	131.0949	$C_6H_{13}NO_2$	2.59	0.34	99	112.9856	1	N.D.	Leucine/Isoleucine II	Amino acid	*Cicer arietinum*/ *Sesamum indicum*	Fabaceae /Pedaliaceae	[21,10]	+	+

13	5.82	180.0668	181.0745	$C_9H_{11}NO_3$	1.73	0.31	96	163.097	5	265	Tyrosine*	Amino acid	*Cicer arietinum*/ *Sesamum indicum*	Fabaceae /Pedaliaceae	[21,10]	+	+
14	6.01	130.087	131.0949	$C_6H_{13}NO_2$	2.98	0.39	86	112.9856	1	N.D.	Leucine/Isoleucine III	Amino acid	*Cicer arietinum*/ *Sesamum indicum*	Fabaceae /Pedaliaceae	[21,10]	+	+
15	6.01	611.1444	612.152	$C_{20}H_{32}N_6O_{12}S_2$	0.25	0.016	98	306.0731, 305.0671, 128.0366	8	N.D.	Oxidized Glutathione (glutathione disulfide)	Peptide	*Sesamum indicum*	Pedaliaceae	[10]	+	+
17	7.75	171.0293	172.0372	$C_7H_8O_5$	3.2	0.6	84	127.0402	4	230	(-)-3-dehydroshikimic acid	Organic acid	*Sesamum indicum*	Pedaliaceae	[10]	+	+
18	7.87	191.0563	192.0634	$C_7H_{12}O_6$	−0.77	−0.15	100	147.0665, 129.0556, 101.0608	2	N.D.	Quinic acid I	Organic acid	*Sesamum indicum*	Pedaliaceae	[10]	+	+
19	8.18	191.0562	192.0634	$C_7H_{12}O_6$	−0.33	−0.06	99	147.0660, 129.0556, 101.0607	2	N.D.	Quinic acid II	Organic acid	*Sesamum indicum*	Pedaliaceae	[10]	+	+
22	9.20	164.0718	165.0790	$C_9H_{11}NO_2$	−0.26	0.0	100	147.049	5	N.D.	Phenylalanine*	Amino acid	*Sesamum indicum*	Pedaliaceae	[10]	+	+
23	10.61	218.1031	219.1107	$C_9H_{17}NO_5$	1.33	0.3	98	146.0819	2	N.D.	Pantothenic acid (Vit B5)	Organic acid	*Cicer arietinum*/ *Sesamum indicum*	Fabaceae /Pedaliaceae	[21,10]	+	+
29	13.05	203.0834	204.0906	$C_{11}H_{12}N_2O_2$	−1.94	−0.4	87	142.0663, 116.0507	7	277	Tryptophan*	Amino acid	*Cicer arietinum*	Fabaceae	[21]	+	+
34	14.49	175.0612	176.0685	$C_7H_{12}O_5$	0.19	0.03	100	115.0402	2	N.D.	Isopropylmalic acid	Organic acid	*Cicer arietinum*/ *Sesamum indicum*	Fabaceae /Pedaliaceae	[21,10]	+	+
77	24.05	187.0979	188.1049	$C_9H_{16}O_4$	−1.75	−0.33	99	125.097	2	N.D.	Azelaic acid	Organic acid	*Cicer arietinum*/ *Sesamum indicum*	Fabaceae /Pedaliaceae	[21,10]	+	+

[a] Detected ions were $[M-H]^-$.* Identification confirmed by comparison with standards; N.D., below 5 mAU or masked by compound with higher signal. Isomers are denoted with letter codes I, II, etc.
